# Hygiene Hypothesis as the Etiology of Kawasaki Disease: Dysregulation of Early B Cell Development

**DOI:** 10.3390/ijms222212334

**Published:** 2021-11-15

**Authors:** Jong-Keuk Lee

**Affiliations:** Asan Medical Center, Asan Institute for Life Sciences, University of Ulsan College of Medicine, Seoul 05505, Korea; cookie_jklee@hotmail.com; Tel.: +82-2-3010-4142

**Keywords:** Kawasaki disease, hygiene hypothesis, B cells, intravenous immunoglobulin

## Abstract

Kawasaki disease (KD) is an acute systemic vasculitis that occurs predominantly in children under 5 years of age. Despite much study, the etiology of KD remains unknown. However, epidemiological and immunological data support the hygiene hypothesis as a possible etiology. It is thought that more sterile or clean modern living environments due to increased use of sanitizing agents, antibiotics, and formula feeding result in a lack of immunological challenges, leading to defective or dysregulated B cell development, accompanied by low IgG and high IgE levels. A lack of B cell immunity may increase sensitivity to unknown environmental triggers that are nonpathogenic in healthy individuals. Genetic studies of KD show that all of the KD susceptibility genes identified by genome-wide association studies are involved in B cell development and function, particularly in early B cell development (from the pro-B to pre-B cell stage). The fact that intravenous immunoglobulin is an effective therapy for KD supports this hypothesis. In this review, I discuss clinical, epidemiological, immunological, and genetic studies showing that the etiopathogenesis of KD in infants and toddlers can be explained by the hygiene hypothesis, and particularly by defects or dysregulation during early B cell development.

## 1. Clinical Characteristics

Kawasaki disease (KD) is an acute systemic vasculitis that occurs predominantly in children under 5 years of age [1]. It is characterized by prolonged fever that is unresponsive to antibiotics; polymorphous skin rash; erythema of the oral mucosa, lips, and tongue; erythema of palms and soles; bilateral conjunctival injection; and cervical lymphadenopathy [2]. KD is often complicated by coronary artery lesions, with approximately 15–25% of untreated children with KD developing coronary artery aneurysm (CAA) [3], making the disease the leading cause of acquired heart disease among children in developed countries. Although treatment with high-dose intravenous immunoglobulin (IVIG) plus aspirin reduces inflammation and the incidence of CAAs markedly, 3–5% of treated children with KD develop CAA, and 1% develop giant aneurysms [4,5].

## 2. Etiology of KD

Since KD was first reported in 1967 by the Japanese pediatrician Tomisaku Kawasaki [2], many studies have tried to identify the underlying cause, with no success. The etiology of KD remains unknown. It is proposed that KD is likely to result from an exaggerated immune response to an environmental or infectious trigger in genetically susceptible children [6,7]. Three hypotheses have been proposed to explain the etiology of KD (Table 1); however, two of these, the infection and autoimmunity hypotheses, seem inappropriate (as explained below).

### 2.1. KD Cannot Be Explained by the Infection Model

Multiple infectious agents have been suspected to cause KD, including bacteria, rickettsia, viruses, and even dust mite antigens [8]. However, no culture- and serology-based studies have identified the infectious agent responsible to date, and none of the proposed etiologic agents has been confirmed [9]. Furthermore, there is no evidence of person-to-person spread, and KD does not respond to antibiotics [10], indicating that the infection model does not explain the etiology of KD. Therefore, infections may be modulating factors rather than the cause of KD [6]. It is more likely that KD is caused by an immunological reaction to either a ubiquitous microorganism or some other environmental agents. 

### 2.2. KD Cannot Be Explained by the Autoimmunity Model

The autoimmunity hypothesis was also investigated as an etiology of KD [11,12]. However, KD is vasculitis with a low rate of recurrence, which does not fit the pattern of autoimmune disorders. Furthermore, KD patients lack a family history of autoimmune diseases [11,12]. Moreover, serological analyses have failed to demonstrate the consistent presence of disease-specific autoantibodies [12]. Therefore, KD is unlikely to be an autoimmune disease. 

### 2.3. KD Can Be Explained by the Hygiene Hypothesis

The hygiene hypothesis was initially proposed to explain the etiology of allergic diseases [13]. The hygiene hypothesis explains that delayed exposure to infectious agents triggers the development of allergic diseases by suppressing the natural development of the immune system. The link between reduced exposure to the biological environment during childhood and increased risk of immune system diseases is consistent with the hygiene hypothesis. This hypothesis can explain diseases such as asthma, allergies, and KD, the incidences of which have increased significantly since the 1960s [6]. The hygiene hypothesis as the etiology of KD [14,15] suggests that a lack of exposure to ubiquitous microbes in early life is associated with development of KD. However, evidence-based mechanisms explaining KD development remain absent. In this review paper, I will discuss the evidence supporting the hygiene hypothesis as the primary etiology of KD, focusing on the role of B cell development and B cell function.

## 3. Epidemiological Evidence for the Hygiene Hypothesis as the Pathogenesis of KD

### 3.1. The Incidence of KD in East Asia Has Been Increasing Since the First Case in 1961

**Figure 1:**
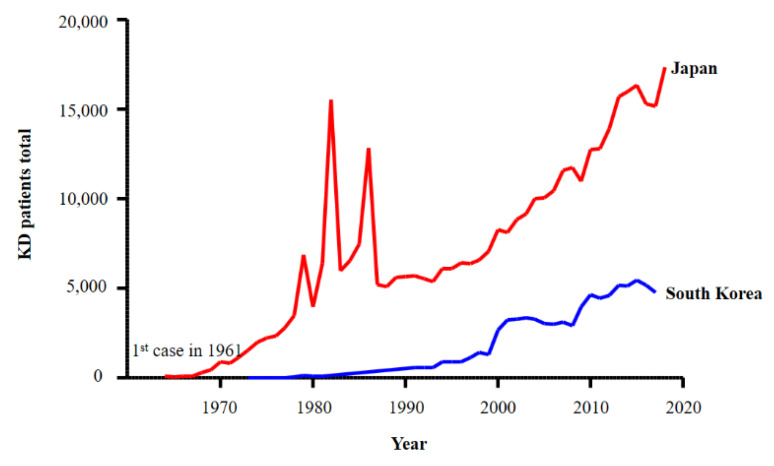
The incidence of Kawasaki disease (KD) in Japan and South Korea has increased continuously since 1961. This graph is based on data collected during nationwide surveys in Japan [1,22,23,24,25,26,27,28,29,30,31,32,33,34,35] and South Korea [36,37,38,39,40,41,42,43]. Three nationwide epidemics were observed in Japan (1979, 1982, and 1986).

In 1961, Dr. Kawasaki observed the first case of an unusual illness manifesting as fever and rash [10]; in 1967, he reported 50 cases in the Japanese Journal of allergy [2]. Similar cases were reported subsequently in South Korea [16], Hawaii in the USA [17], and Taiwan [18]. Since then, the global incidence of KD has been increasing continuously. Although cases of KD have been reported in all ethnicities, it is most common in children of Asian descent [19]. Japan has the highest incidence of KD (308/100,000 children aged <5 years), followed by South Korea (217/100,000 children aged <5 years), China, and Taiwan [20,21]. The incidence in Northeast Asian countries is 10–30 times higher than that in North America and Europe. Japan and South Korea perform nationwide epidemiologic surveys every 2 and 3 years, respectively [20,21]. The data confirm that the incidence of KD in both countries is increasing (Figure 1). This continuous increase since 1961 suggests that the cause, or trigger factor(s), is associated with modern living environments. 

### 3.2. Improved Socioeconomic Environments Increase the Incidence of KD

A large longitudinal survey of newborns (*n* = 41,872) in Japan showed that better socioeconomic environments, such as higher income, smaller family size (less children in household), and urbanized life, are significantly associated with the increased incidence of KD (Table 2) [44]. Data from Japan [44,45], Korea [42], and Taiwan [46] showed that the incidence of KD in rural areas is lower than that in urban areas (probably due to more sanitary conditions in the latter). Taken together, the data suggest that better socioeconomic environments and sanitation are preconditions for KD, supporting the hygiene hypothesis as the etiology of KD. 

### 3.3. Vaccination Protects against KD

There is no causal association between vaccination and the development of KD [47,48,49,50,51]. However, data from 1,721,186 children aged 0–6 years suggest that the rate of verified KD is significantly lower during the first 1–42 days post-vaccination (rate ratio = 0.50; 95% confidence interval (CI] = 0.27–0.92) or 8–42 days post-vaccination (rate ratio = 0.45; 95% CI = 0.22–0.90) than during periods when children were unexposed to vaccine [49], indicating that childhood vaccination is associated with a transient decrease in the incidence of KD. The protective effect of vaccination suggests that B cell immunity may play a role in development of KD, again supporting the hygiene hypothesis. 

**Table 2:** Epidemiological risk factors for Kawasaki disease (KD).

Factor	Effect on KD	References
Better socioeconomic environment: - high income - smaller family size - urbanized life	Risk	[44]
Urban areas	Risk	[42,44,45,46]
Gestational age and body weight at birth: - pre-term delivery - lower body weight at birth	Risk	[32]
Vaccination	Protective	[49]
Breastfeeding	Protective	[52,53,54]

KD, Kawasaki disease.

### 3.4. Breastfeeding Protects against KD

Studies from Japan [52], Germany [53], and China [54] show that, compared with formula feeding, breastfeeding has a protective effect against development of KD (Table 2). The protective effect of breastfeeding is mainly due to colostrum [52]. Breastfeeding and breastmilk, including colostrum, are thought to help the immune system mature by establishing the intestinal microbiota [55,56]. Therefore, it is possible that immune substances such as secretary antibodies in either breastmilk or colostrum may play a role (either directly or indirectly) by facilitating early immune responses in infants; these responses may protect against the development of KD. 

## 4. Insufficient B Cell Immunity Is Crucial for the Development of KD

### 4.1. The Peak Incidence of KD Overlaps with the Lowest Level of B Cell Immunity

**Figure 2:**
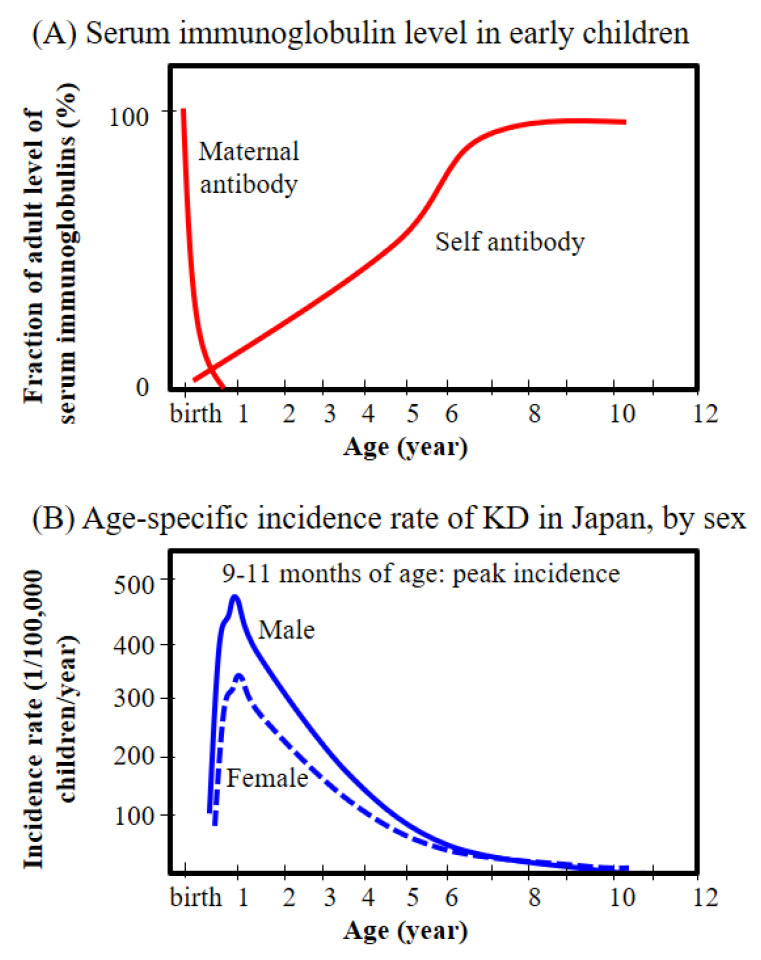
Immunoglobulin levels in children (**A**) and age-specific incidence rates of Kawasaki disease (KD) in Japan (**B**). The immunoglobulin levels in children depicted above were obtained from an immunology textbook [58]. The age-specific incidence rate of Kawasaki disease (KD) by sex was simplified from epidemiological data of Japan [33].

KD affects predominantly young children; indeed, 85% of cases occur in children younger than 5 years old [20,57]. Japanese data show that the peak incidence occurs at 6 months to 2 years of age, particularly between 9 and 11 months [33], which coincides with periods of low immunoglobulin G (IgG) levels due to waning maternal antibody levels and low active immune development (Figure 2). The fact that KD is rare in children aged less than 6 months supports the notion that passive immunity conferred by maternal antibodies protects against KD development. In addition, the incidence in children older than 5 years (when they have fully developed B cell immune responses) falls markedly. Furthermore, KD is virtually absent in adults. In comparison with the general population, patients with KD tend to be born earlier and have smaller birth weights [32], suggesting that immature or insufficient immune development due to pre-term birth and low birth weight may make very young children more susceptible. Therefore, the age-dependent incidences of KD observed in epidemiological studies suggest that poor antibody-mediated B cell immunity increases the risk of KD. 

### 4.2. KD Occurs in Children and Adults with Primary and Secondary Immunodeficiencies

KD has been described as a complication of diverse primary and secondary immunodeficiency disorders. Nineteen studies were found reporting forty KD patients with immunodeficiencies [59]. The most convincing evidence that immunodeficiency predisposes a person to the development of KD comes from a study of adults with KD. KD is rare in adults; about one-third of adult KD cases are associated with HIV infection [59]. More than 20 HIV-infected patients with KD have been reported. Reported cases of KD in immunodeficient children and in adults with HIV infection suggest that immunodeficiency disorders may lead to persistent inflammation and subsequent development of KD. 

### 4.3. Patients with KD Have a High Risk for Allergic Diseases and Vice Versa

**Figure 3:**
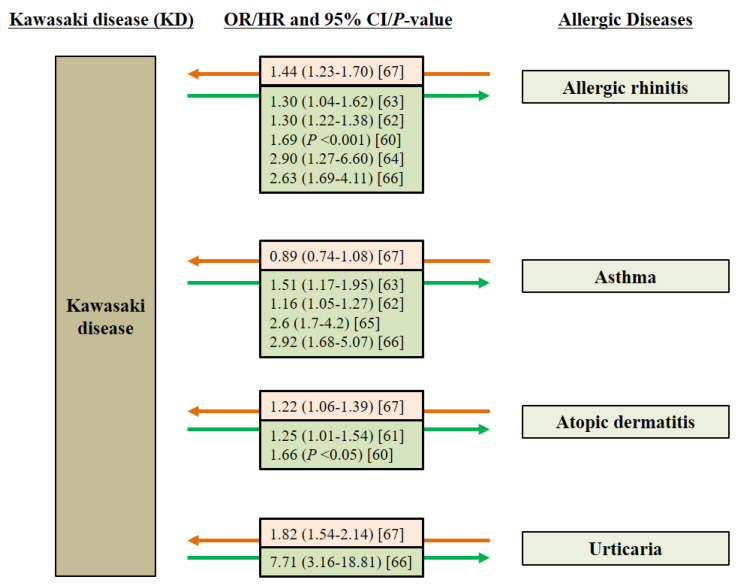
Increased risk of Kawasaki disease (KD) in children with common allergic diseases and vice versa. CI, confidence interval; HR, hazard ratio; OR, odds ratio.

In Japan, the incidence of atopic dermatitis and allergic rhinitis is significantly higher (~1.7 times) in KD patients than in controls [60]. Children with KD also tend to have a higher risk of susceptibility to atopic dermatitis, allergic rhinitis, asthma, and urticaria [61,62,63,64,65,66]. In addition, children with allergic diseases are significantly more likely to develop KD [67] (Figure 3). These results suggest that KD shares common pathogenetic mechanisms with allergic diseases. Patients with KD are more likely to live in environments with low exposure to environmental allergens, such as urban areas. 

## 5. Immunological Evidence from Patients with KD Demonstrates that B Cell Immunity Is Crucial for the Etiopathogenesis of KD

### 5.1. Severe Inflammatory Responses in Patients with KD

Severe inflammation was observed in patients with acute KD, with a significant increase in WBC count, mainly neutrophils (especially immature) and monocytes [68,69]. By contrast, lymphocytes were decreased. Interestingly, B cells were significantly increased in patients with acute KD (by approximately 1.3 times compared with healthy controls), although T cells and NK cells were significantly reduced [68] (Table 3). The finding that T cells were significantly reduced and B cells were significantly increased during the acute phase of KD was also reported in another study [70]. In the course of KD, the number of neutrophils is the highest during the acute phase and decreases gradually during the subacute and convalescent phases. On the contrary, the number of lymphocytes is the lowest during the acute phase but increases gradually after the subacute and convalescent phases [71]. A study of gene expression using blood samples from patients with KD also observed a decrease in expression of lymphocyte-related genes [72]. Therefore, the significant increase of neutrophils, monocytes, and B cells in acute KD suggests that these cell types play a crucial role in pathogenesis. 

### 5.2. Low IgG Levels in Patients with KD

Studies from the USA [73], China [74], Taiwan [75], and Japan [76] report that serum levels of IgG in patients with KD before IVIG treatment are significantly lower than those in healthy children of the same ages. In particular, a Japanese study [76] showed that serum IgG levels prior to IVIG treatment were significantly lower in all age groups of KD patients (mean z-score for IgG level = −0.60; *n* = 418). However, after IVIG treatment, the IgG level in the blood was very high (mean z-score = 9.60; *n* = 418). In addition to IgG, the levels of other immunoglobulin isotypes (IgA, IgM, IgD, and IgE) also increased after IVIG treatment [77,78], suggesting that polyclonal B cells may be activated during recovery after IVIG treatment. Other reports show that low levels of IgG in patients with KD before or after treatment are risk factors for coronary artery lesions [79,80]. In addition, a report [76] suggests that low IgG levels in KD patients before IVIG treatment are significantly associated with nonresponse to IVIG. However, this was not a significant finding in Korean patients with KD [81]. Therefore, it is still uncertain whether low levels of IgG affect responses to IVIG treatment. The reduction in IgG levels in patients with KD prior to IVIG treatment suggests that a cleaner environment may be associated with the development of KD. Furthermore, compared with febrile controls or KD patients after IVIG treatment, analysis of the B cell receptor (BCR) repertoire using next-generation sequencing (NGS) identified lower B cell diversity during the acute phase of KD [82,83]. Thus, during the acute phase of KD, B cell counts are significantly increased [68,84], but IgG levels are decreased [69,74], with lower B cell diversity [82,83], suggesting that the B cells are nonfunctional, immature, and/or not activated in acute KD.

### 5.3. High IgE and Eosinophil Levels in Patients with KD

In contrast to low IgG levels, patients with KD show significantly elevated IgE levels [61,78,81,85,86,87,88]. In particular, elevated IgE levels were reported in 62% of patients with KD [88] compared with normal values in the same age group, with higher IgE levels in younger patients (80% in 1 year olds vs. 69% in 1–3 year olds vs. 44% in >3 year olds). The high IgE levels in patients with KD were maintained throughout all clinical phases (acute, subacute, and convalescent) [81]. The role of elevated IgE levels in patients with KD is still unknown. However, it is reported that high IgE levels in patients with KD are not associated with any other inflammatory biomarkers or with a clinical phenotype of KD [81], suggesting that elevated IgE is not involved in the pathological mechanism. In addition, germ-free or antibiotic-treated mice have high IgE levels [89,90], and the IgE in these mice is produced by immature B cells [91]. The hygiene hypothesis for allergic diseases associated with high IgE levels [13] may also explain the high IgE levels in patients with KD; better hygiene in infancy and young childhood may lead to defects or dysregulation in B cell development, resulting in low amounts of IgG and high amounts of IgE produced by immature B cells, thereby increasing susceptibility to KD. This concept is supported by the finding that patients with KD are also highly susceptible to allergic diseases [60,63]. In addition, the incidence of atopic dermatitis among children with KD is nine times higher than that of normal controls, and serum IgE levels are significantly higher in KD patients than in healthy individuals [92]. Furthermore, compared with those in female children in Korea, IgE levels in males are 1.5-fold higher [93], which may explain the approximately 1.5-fold higher incidence of KD in males compared with females [1,43,94]. Moreover, eosinophils are significantly elevated in KD both before and after IVIG treatment, and eosinophilia after IVIG treatment shows an inverse correlation with IVIG resistance [95]. Several studies report eosinophilia in patients with KD, including the original report by Dr. Kawasaki [10,71,95,96,97]. The strong association between KD and atopic dermatitis, allergy, elevated serum IgE levels, and eosinophilia indicates that KD and allergic diseases may share the same underlying mechanism of etiopathogenesis. 

## 6. Efficacy of IVIG Treatment Demonstrates That B Cell Development and Activation Are Crucial for the Pathogenesis of KD 

### 6.1. Lower IgG Levels in Patients with KD Correlate with Worse Clinical Outcomes, and IVIG Is the Standard Therapy for KD

Low IgG levels before or after IVIG treatment correlate with worse clinical outcomes, including more severe inflammation, coronary artery abnormalities, and IVIG resistance [76,79,80,98,99]. Therefore, it is speculated that the low IgG level plays an important role in occurrence and prognosis of KD. High-dose IVIG (2 g/kg) plus aspirin is a very effective treatment for patients with KD.

### 6.2. The Therapeutic Effect of IVIG Is Not Likely Due to Passive Protection or Anti-Inflammatory Effects

IVIG (polyclonal IgG pooled from the serum of thousands of donors) is used to provide passive protection to those with primary immunodeficiency diseases and to treat severe inflammatory or autoimmune disorders [100]. The therapeutic mechanism by which IVIG ameliorates KD is unknown. Most people believe that the anti-inflammatory role of IVIG may be the key. However, this is unlikely to be the case for KD, because steroids, which have anti-inflammatory activity, have little therapeutic effect [101,102,103,104]. In addition, the anti-inflammatory activity of IVIG is dependent on glycosylation of the Fc portion of IgG [105]. Genes associated with glycosylation of IgG appear to play no role in KD or in IVIG resistance (our own unpublished data), indicating that IVIG does not act as an anti-inflammatory agent in KD. These findings indicate that the anti-inflammatory activity of IVIG may not play a role in its effect in KD. Another possible therapeutic mechanism of IVIG in KD is the provision of passive protection against unknown trigger antigens. However, it is difficult to confirm this possibility because the cause of KD remains unknown. If IVIG is given earlier (e.g., within 4 days of clinical onset), the therapeutic effect is lower than that observed when IVIG is given later than 5 days post onset [106]. This indicates that IVIG is unlikely to provide passive protection (i.e., neutralizing unknown antigens). 

### 6.3. The Therapeutic Effect of IVIG Is Likely Due to the Activation of Endogenous B Cell Development and Function

The most likely explanation for the therapeutic effect of IVIG in KD is that IVIG may play a crucial role in activating B cell development and function. This concept is supported by the following facts. First, in most patients with KD, fever subsides after a few weeks, even without special treatment [57]. However, if not treated properly, the sequelae of vasculitis can lead to CAA in about 15–25% of children, leading to myocardial infarction and death [4,107]. Earlier IVIG treatment (within 4 days after onset, compared with conventional treatment given at 5–7 days after onset) has a lower therapeutic effect and results in higher rates of IVIG resistance [106]. In addition, it is reported that serum IgG levels in patients with KD are further elevated at the time of discharge [76], indicating that elevated IgG levels may be due to endogenous B cell development and activation. Furthermore, responses to IVIG treatment in those with KD are associated with sialylation levels of endogenous, not therapeutic exogenous, IgG [108], supporting the notion that stimulation of host B cells by exogenous immunoglobulin (i.e., IVIG) is crucial for recovery. As mentioned previously [12], the low recurrence rates of KD (3–4% in Japan [109]) and lack of immune defects after KD also suggest that endogenous B cell activation or immunizing events occur during both development of KD and recovery from KD. In recurred KD, the duration of fever is shorter than that in its first occurrence [110,111], supporting the role of immunizing events in the course of KD. All of these results strongly suggest that endogenous B cell development and activation play a crucial role in the therapeutic effects of IVIG in patients with KD. 

## 7. Genetic Evidence Indicates That Defects in Early B Cell Development May Be Critical for the Etiopathogenesis of KD

### 7.1. Genetic Susceptibility Affects the Incidence of KD

The incidence of KD is extremely high in East Asian populations, especially among the Japanese, South Koreans, Taiwanese, and Chinese [19,21]. In Japan, the incidence is 10–20 times higher than those in Western countries [112,113]. A survey in the United States, which examined a multiracial population, also showed the highest incidence of KD among Asians, with the incidence decreasing in Africans and Caucasians [114,115]. Therefore, racial differences in the incidence of KD suggest that genetic susceptibility may be an important factor in the high incidence of KD in East Asian populations. Brothers and sisters of children with KD are at 10-fold higher risk of developing KD compared to the general population [116]. In addition, the incidence of KD in children born to parents with a history of KD is twice as high as that in the general population [117]. All of the findings strongly suggest that genetic factors play a role in the development of KD.

### 7.2. KD Susceptibility Genes Are Involved in Early B Cell Development and Function

Recently a series of genome-wide association studies (GWAS) identified several KD susceptibility genes, including *BLK, CD40, FCGR2A, IGHV*, and *BCL2L11* (Table 4). Interestingly, all KD susceptibility genes are related to B cells (genes involved in early B cell development and B cell function), suggesting that B cell immunity is crucial for the development of KD. Among the KD susceptibility genes, risk alleles of genes associated with early B cell development (*BLK* and *BCL2L11* genes) are linked to decreased gene expression. By contrast, risk alleles of downstream genes associated with B cell function (*CD40*, *FCGR2A*, and *IGHV* genes) are linked to enhancement of B cell function (Table 5). We do not know how KD susceptibility genes affect development of KD. Although innate immunity plays a central role in host defense against viral and bacterial infections, the KD susceptibility genes identified by GWAS are not related to innate immunity. Therefore, viral and bacterial infections are unlikely to be involved in development of KD. In addition, there was no significant difference in the frequency of risk alleles of KD susceptibility genes among different ethnic populations [118], suggesting that the high incidence of KD in East Asians is not due to differences in the frequency of risk alleles of susceptibility genes but more likely due to factors such as differences in living environment.

### 7.3. Reduced Expression of Risk Alleles of BLK and BCL2L11 Genes in KD Suggests That the Development of KD Is Due to Defects or Dysregulation of Early B Cell Development

The B lymphoid tyrosine kinase (*BLK*) gene is associated most significantly with KD (Table 4). *BLK* is an Src family kinase expressed mainly in B cells, and it is involved in BCR signal transduction and B cell development [126,127,128]. Specifically, signaling through *BLK* plays an important role in transmitting signals via the BCR expressed on the surface of B cells, which promotes differentiation from the pro-B to pre-B cell stage, as well as transducing signals that trigger cell cycle arrest and apoptosis downstream of the BCR [129]. *BLK* specifically binds and phosphorylates CD79A (Igα) at Tyr-188 and Tyr-199, as well as CD79B (Igβ) at Tyr-196 and Tyr-207. *BLK* also phosphorylates the immunoreceptor tyrosine-based activation motif (ITAM) of the IgG receptor *FCGR2A*, *FCGR2B,* and *FCGR2C*, which are expressed on B cells [127]. *BLK* expression is observed mainly in B cells during early development [130]. In particular, *BLK* expression in mice is first observed in cycling late pro-B cells, and expression continues throughout B cell development, before being downregulated in plasma B cells [131]. In addition, *BLK* gene expression is associated with age; it is expressed at high levels in the young and at low levels in the old [132]. These findings indicate that *BLK* plays a crucial role in early B cell development, particularly at a young age. The *BLK* locus is associated with various immune diseases, including rheumatoid arthritis, systemic lupus erythematosus, systemic sclerosis, and KD [124,133,134,135]. Risk alleles of the *BLK* gene associated with systemic lupus erythematosus (SLE) [136] and KD [125] are linked to reduced expression of *BLK*. These findings suggest that reduced expression of *BLK* results in defects or dysregulation of B cell development, particularly in young children, making these children more susceptible to KD. 

Bcl-2-like protein 11 (*BCL2L11*), a proapoptotic BH3-only protein within the Bcl-2 family, is highly expressed in the immune system, including in bone marrow and lymph nodes. High expression of *BCL2L11* promotes lymphocyte apoptosis, whereas lack of *BCL2L11* can increase the survival of autoreactive B cells and T cells [137,138,139]. This indicates that *BCL2L11* plays a critical role in regulating lymphocyte homeostasis. The *BCL2L11* locus is associated with several traits and immune diseases, such as the percentages of eosinophils and neutrophils, lymphocyte counts, B cell chronic lymphocytic leukemia, asthma, and KD [122,140,141,142,143,144]. In the case of KD, an intronic single-nucleotide polymorphism (SNP) (rs3789065) within the *BCL2L11* gene is significantly associated with KD at the genome-wide significance level in IVIG responders but not in nonresponders [122]. Interestingly, the KD-associated intronic SNP (rs3789065) is associated with serum concentration of inflammation-associated complement component 3 peptide [145], and a risk allele (C allele) of the KD-associated SNP (rs3789065) results in significantly reduced expression of the *BCL2L11* gene in human peripheral blood monocytes (*p* = 4.23 × 10^–20^) [132]. Taken together, these findings suggest that allele-specific reduction in *BCL2L11* increases susceptibility to KD, particularly in IVIG responders. Reduced expression of risk alleles of both *BLK* and *BCL2L11* genes indicates that KD may be caused by defects in early B cell development. In contrast to the *BLK* and *BCL2L11* genes, other B cell–related KD susceptibility genes (*CD40*, *FCGR2A*, and *IGHV*) show opposite patterns, that is, increased expression or enhanced functions (Table 5). Although the effects of these genes on KD susceptibility are weaker than those of *BLK* and *BCL2L11* genes, the associations between highly expressed or activated alleles and KD strongly suggest that B cells or endogenous immunoglobulins play an active role in its pathogenesis. However, it remains unclear how these genes affect KD susceptibility. 

### 7.4. Male-Dominant Incidence of KD May Be Due to Male-Specific Susceptibility through the FCGR2A Gene

*FCGR2A* encodes a low-affinity Fc gamma receptor that binds to the Fc portion of IgG. This protein is a cell surface receptor expressed on phagocytic cells such as macrophages and neutrophils and is involved in the phagocytosis and clearing of immune complexes. Higher expression of *FCGR2A* was found in monocytes of patients with KD [146]. When ligated by immune complexes, *FCGR2A* transduces activating signals to immune cells via the ITAM in the cytoplasmic domain. Human B cells also express four IgG receptors: FcγRIIa, FcγRIIb1, FcγRIIb2, and FcγRIIc. FcγRII receptors, including FcγRIIa, are phosphorylated by BLK. Co-ligation of either FcγRII isoform with the BCR abrogates B cell activation [127]. Soluble FcγRII receptors, which have been identified in biological fluids from mice and humans, are produced either by alternative splicing of the exon encoding the transmembrane region of the receptor or by proteolytic cleavage at the cell membrane. They inhibit B cell proliferation and immunoglobulin production [147]. Therefore, *FCGR2A* may act as a KD susceptibility gene by abrogating B cell activation via co-ligating FcγRIIa and the BCR or by inhibiting B cell proliferation and immunoglobulin production via soluble FcγRIIa. The KD-associated *FCGR2A* SNP (rs1801274; A/G, p.His167Arg, previously assigned as p.His131Arg) is associated with autoimmune diseases such as ankylosing spondylitis [148], SLE [149], inflammatory bowel disease (IBD) [150,151,152], and KD [123]. The A allele of the *FCGR2A* SNP (rs1801274) plays a role in susceptibility to IBD and KD, whereas the G allele is a risk allele for SLE [149], which is more common in females (Table 6). This variant (rs1801274) is also reported to affect binding affinity to IgG2. The A allele of *FCGR2A*, which encodes a histidine residue, binds to IgG2 with high affinity, whereas the G allele, which encodes an arginine residue, shows little or no binding affinity to IgG2 [153,154,155]. These findings suggest that the functional SNP (rs1801274, encoding p.His167Arg) in the *FCGR2A* gene has a disease-specific effect on susceptibility. In addition, the expression of *FCGR2A* mRNA is lower in the monocytes of human males than in those of females [132]; the same is true for other white blood cells (GTEx database: https://www.gtexportal.org/home/gene/FCGR2A) (accessed on 11 Jan. 2021). Furthermore, the association between the *FCGR2A* SNP (rs1801274, the A risk allele encoding histidine) and KD is significant in males only [156], particularly in those aged less than 1 year [157]. These findings suggest that the higher susceptibility of males to KD (approximately 1.5-fold higher than females) [57] may be explained by sex-specific effects of the *FCGR2A* gene (particularly lower expression of *FCGR2A* in males) and a male-specific association between a high affinity functional SNP (rs1801274, A allele encoding histidine) and KD in children less than 1 year old. The different effects of the *FCGR2A* gene in SLE and KD suggest that development of KD is due to insufficient B cell immunity, whereas development of SLE is due to autoimmune activation (activation of B cell immunity). The opposite effects of the *FCGR2A* gene in KD and SLE are consistent with the sex-dependent incidence of KD (1.5-fold higher in males) [57] and SLE (9-fold higher in females) [158]. However, estrogen protects B cells from BCR-mediated apoptosis [159] and increases IgG and IgM production by peripheral blood mononuclear cells in both men and women [160]. In addition, a gene locus (rs781858752) in the immunoglobulin heavy chain (IGH) region is significantly associated with estradiol levels in men (*p* = 7.6 × 10^−15^) and affects the expression of IGHV3-9 and IGHV1-8 in the liver [161]. Furthermore, an intronic SNP (rs12613243) located in the KD susceptibility gene *BCL2L11* is strongly associated with sex hormone binding to globulins (*p* = 9 × 10^−9^), which affects testosterone levels [161]. Therefore, we cannot exclude a possible role of sex-hormone-dependent B cell development and function in the sex-dependent incidence of KD.

**Figure 4:**
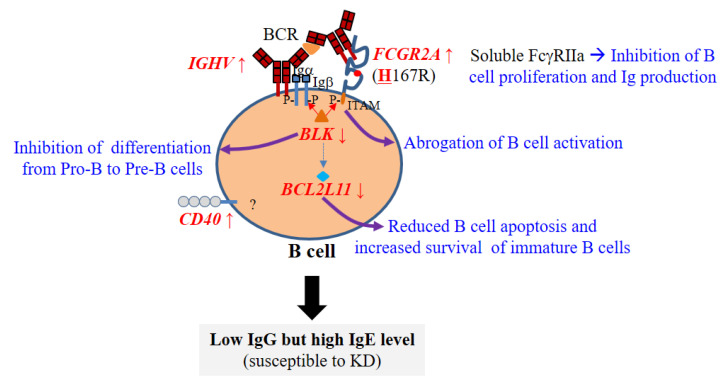
Genetic studies support early B cell–mediated pathogenesis of KD. The effect of risk alleles of KD susceptibility genes on mRNA expression is shown by arrows (↑: upregulation and ↓: downregulation). BCR, B cell receptor; KD, Kawasaki disease.

As mentioned above, two main KD susceptibility genes (*BLK* and *BCL2L11* genes) in both males and females are involved in early B cell development (Figure 4). The male-specific association between *FCGR2A* and KD, coupled with lower *FCGR2A* gene expression in males, may be one reason why males are more susceptible to KD. Furthermore, the major KD susceptibility genes (*BLK* and *BCL2L11*) are involved mainly in early B cell development and signaling pathways [163,164]. However, genes involved in mature B cell signaling (e.g., *CD19*, *CD22*, *CD81*, *LYN*, *PTPN6 (SHP-1)*, *TNFSF13B (BAFF)*, *TNFRSF13C (BAFF-R)*, *TNFSF13 (APRIL)*, *TNFRSF13B (TACI)*, and *TNFRSF17 (BCMA)*) are not associated with KD (our own unpublished GWAS data). This suggests that defects or dysregulation of early B cell development, rather than mature B cells, in young children, is crucial for the pathogenesis of KD. Taken together, genetic evidence strongly supports the hygiene hypothesis, particularly defective or dysregulated early B cell development, as the pathogenetic mechanism underlying KD.

## 8. Conclusions and Proposed Mechanism Underlying the Etiopathogenesis of KD 

**Figure 5:**
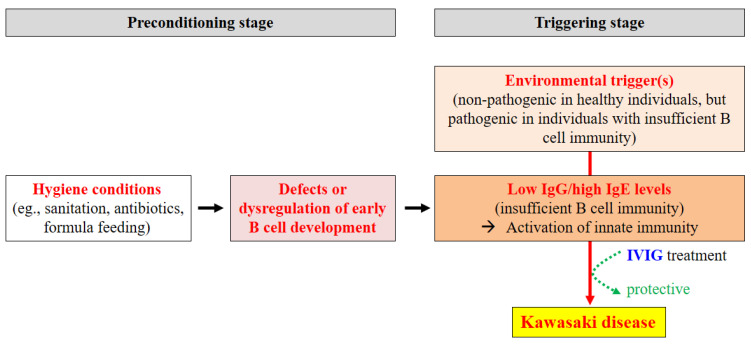
Proposed mechanism underlying the hygiene-hypothesis-driven etiopathogenesis of KD. IVIG, intravenous immunoglobulin.

Epidemiological, immunological, and genetic evidence supports the hygiene hypothesis as the etiology of KD, mainly due to defects or dysregulation of early B cell development (Figure 5). Epidemiological observations suggest that modern living environments with hygienic conditions are a major cause of KD. Lack of exposure to pathogens and other antigens in early life leads to defective or dysregulated early B cell development due to a lack of immunological challenges. In addition, significantly lower IgG levels and elevated IgE levels in patients with KD indicate insufficient B cell immunity, which can lead to sensitization to foreign antigens and activation of innate immune responses. Insufficiency of B cell immunity in KD is supported by the fact that IVIG is a very effective therapy for patients with KD. The possible pathogenetic mechanism underlying KD might be split into the preconditioning stage and the triggering stage. Insufficient B cell immunity occurs during the preconditioning stage; then, unknown environmental triggers activate innate immune responses during the triggering stage (Figure 5). Identification of trigger factors, such as infectious agents and/or environmental antigens that activate innate immunity, will be not easy because most trigger factors might be nonpathogenic in healthy individuals. This may be the reason why we have not yet identified causative agents, although several potential trigger agents have been proposed, including microbiomes (fungal, bacterial, and viral), climate factors, lead poisoning, etc. In addition, attempts to identify BCRs specific for KD would likely fail because there are too many potential trigger factors, as NGS has revealed.

## Figures and Tables

**Table 1:** Three hypotheses that explain the etiology of KD.

	Infection Hypothesis	Autoimmunity Hypothesis	Hygiene Hypothesis
Pros	Elevation of WBC count, (especially immature neutrophils) Increased levels of inflammatory markers, such as CRP and TNF-α Seasonal variations: a peak in winter and a trough in autumn	Anti-endothelial cell autoantibodies in acute KD High eosinophil count in patients with KD	No KD cases reported before 1961 Peak of incidence when immunoglobulin levels are at the lowest (at 9–12 months of age) Virtual absence of KD in adults Increased incidence in better socioeconomic environments Frequent association of KD with a variety of primary immunodeficiency disorders High IgE and low IgG levels Protective effect of vaccination and breastfeeding Efficacy of IVIG Association with genes involved in B cell development and function (GWAS)
Cons	No evidence for person-to-person transmission No identification of an infectious agent responsible for KD No response to antibiotics No association with genes involved in innate immunity	Low rate of recurrence No concomitant autoimmune diseases No family history of autoimmune diseases	

CRP, C-reactive protein; GWAS, genome-wide association study; IVIG, intravenous immunoglobulin; KD, Kawasaki disease; TNF-α, tumor necrosis factor alpha; WBC, white blood cell.

**Table 3:** Changes in WBC composition during the acute stage of KD.

Cell Type	No. of Samples (Acute KD: Convalescent KD: Healthy Controls)	-Fold Change (Compared with Healthy Controls)		*p*-Value	References
		Acute KD	Convalescent KD		
**WBC (#)**	33:33:25	**1.75** **	1.05	<0.01	[69]
	106:68:22	**1.85** **	1.02	<0.01	[68]
PBMC (#)	106:68:22	0.94	1.08	ns	[68]
Neutrophils (#)	33:33:25	**2.54** **	0.92	<0.01	[69]
Immature neutrophils (#)	33:33:25	**37** **	2.55	<0.01	[69]
Monocytes (#)	33:33:25	1.58	1.06	ns	[69]
	106:68:22	**2.89** **	1.39	<0.01	[68]
Lymphocytes (#):	106:68:22	0.87	1.04	ns	[68]
CD3+ T cells	106:68:22	0.80	1.06	ns	[68]
CD4+ T cells	106:68:22	**0.78** *	0.97	<0.05	[68]
CD8+ T cells	106:68:22	**0.83** *	1.21	<0.05	[68]
CD57+ NK cells	106:68:22	**0.69** *	1.66	<0.05	[68]
CD19+ B cells	106:68:22	**1.31** *	1.06	<0.05	[68]
IgG (mg/dL)	33:33:25	0.95	1.50 **	ns	[69]
CRP (mg/dL)	33:33:25	**39** **	1	<0.01	[69]

#, cell number; CRP, C-reactive protein; ns, not significant; PBMC, peripheral blood mononuclear cell; WBC, white blood cell; Immature neutrophils: 5% of neutrophils in healthy control vs. 26% of neutrophils in acute KD. Significant *P*-values are shown in **bold**. * *p* <0.05; ** *p* <0.01.

**Table 4:** KD susceptibility genes with genome-wide significance, as identified by GWAS (*p* < 5 × 10^−8^).

Gene	SNP	Population	Sample Size (Case:Control)	OR	*p*-Value	References
*BLK*	rs2254546	Japan	1182:4326	1.85	8.2 × 10^−21^	[119]
	rs2736340	Taiwan	883:1657	1.54	9.0 × 10^−10^	[120]
	rs6993775	Korea	915:4553	1.52	2.5 × 10^−11^	[121]
*BCL2L11*	rs3789065	Korea	846:4553	1.42	4.4 × 10^−11^	[122]
*CD40*	rs4813003	Japan	1182:4326	1.41	4.8 × 10^−8^	[119]
	rs1569723	Taiwan	883:1657	1.42	5.7 × 10^−9^	[120]
	rs1883834	Korea	915:4553	1.18	0.003912	unpublished data
*FCGR2A*	rs1801274	Multi-ethnic	1433:7764	1.32	7.4 × 10^−11^	[123]
		Japan	1182:4326	na	1.6 × 10^−6^	[119]
		Korea	915:4553	1.30	5.7 × 10^−5^	[121]
*IGHV*	rs4774175	Japan, Taiwan, Korea	3428:7837	1.20	6.0 × 10^−9^	[124]
	rs6423677 *	Japan	3603:5731	1.25	6.8 × 10^−10^	[124]

GWAS, genome-wide association study; OR, odds ratio; na, not available. * rs6423677 is a nonsynonymous SNP linked with a KD-associated SNP (rs4774175), which was detected by meta-analysis of three East Asian samples (Japan, Taiwan, and Korea).

**Table 5:** Functional effects of risk alleles of KD susceptibility genes.

Gene	SNP	Risk Allele	Functional Effects of Risk Alleles	References
*BLK*	rs2254546 (A/G)	G	Decreased *BLK* mRNA expression	[125]
*BCL2L11*	rs3789065 (C/G)	C	Reduced *BCL2L11* mRNA expression	[126]
*CD40*	rs4813003 (C/T)	C	Enhancement of *CD40* function	[118]
*FCGR2A*	rs1801274 (A/G; H167R *)	A	High-affinity receptor leading to immune activation	[118,123]
*IGHV3-66*	rs6423677 (A/C; p.Cys/Gly)	C	C (risk allele): very high mRNA expression.	[124]

SNP, single-nucleotide polymorphism. * H167R was known previously as H131R.

**Table 6:** Different effects of the risk allele of *FCGR2A* variant rs1801274.

Risk Allele of the FCGR2A Variant (rs1801274; A/G = H167R *)	Effect of Risk Allele on IgG Binding	Disease or Trait Associated with Same Risk Allele	OR or Beta	*p*-Value	References
A allele encoding His (H)	high affinity	AS	1.11	1 × 10^−9^	[148]
		IBD	1.13	9 × 10^−36^	[150]
		IBD	1.12	2 × 10^−38^	[152]
		UC	1.19	1 × 10^−41^	[150]
		UC	1.21	2 × 10^−20^	[151]
		CD	1.08	9 × 10^−11^	[150]
		KD	1.32	7 × 10^−11^	[123]
G allele encoding Arg (R)	little or no affinity	SLE	1.16	1 × 10^−12^	[149]
		Basophil count	0.017 unit increase	3 × 10^−14^	[141]
		Blood FcγRIIa levels	1.24 unit increase	1 × 10^−2102^	[162]

Data was extracted from the GWAS catalog database: https://www.ebi.ac.uk/gwas/ (accessed on 11 January 2021), AS, ankylosing spondylitis; Crohn’s disease, CD; IBD, inflammatory bowel disease; KD, Kawasaki disease; OR, odds ratio; SLE, systemic lupus erythematosus; UC, ulcerative colitis. * H167R was known previously as H131R.

## Data Availability

Not applicable.
